# Protective Effect of Glycomacropeptide on Food Allergy with Gastrointestinal Manifestations in a Rat Model through Down-Regulation of Type 2 Immune Response

**DOI:** 10.3390/nu12102942

**Published:** 2020-09-25

**Authors:** Diana Reyes-Pavón, Daniel Cervantes-García, Luis G. Bermúdez-Humarán, Laura Elena Córdova-Dávalos, Andrés Quintanar-Stephano, Mariela Jiménez, Eva Salinas

**Affiliations:** 1 Department of Microbiology, Basic Science Center, Autonomous University of Aguascalientes, 20131 Aguascalientes, Mexico; nutrois@gmail.com (D.R.-P.); dcervantesga@conacyt.mx (D.C.-G.); lcdavalos@gmail.com (L.E.C.-D.); 2 National Council of Science and Technology, 03940 Mexico City, Mexico; 3 Université Paris-Saclay, INRAE, AgroParisTech, Micalis Institute, 78350 Jouy-en-Josas, France; luis.bermudez@jouy.inrae.fr; 4 Department of Physiology and Pharmacology, Basic Science Center, Autonomous University of Aguascalientes, 20131 Aguascalientes, Mexico; aquinta@correo.uaa.mx

**Keywords:** glycomacropeptide, food allergy model, allergic diarrhea, allergen-specific immunoglobulin, histamine, intestinal inflammation, intestinal histopathology, type 2 immune response

## Abstract

Glycomacropeptide (GMP) is a bioactive peptide derived from milk κ-casein with immune-modulatory and anti-inflammatory properties. Food allergy (FA) is an adverse immune reaction with a broad spectrum of manifestations. Allergen intake induces persistent intestinal inflammation and tissue damage. In this study, the anti-allergic activity of GMP was evaluated using a rat ovalbumin (OVA)-induced FA model with gastrointestinal manifestation. Rats were orally GMP treated from 3 days prior and during FA development. The severity of food anaphylaxis and diarrheal episodes, antibody production and histamine level were measured. Histopathological changes, inflammation and predominant cytokine profile at intestine were analyzed. Oral GMP intake decreased clinical signs and diarrhea severity induced by allergen, with a significant reduction in intestinal edema and expression level of *IL-1β* and *TNF-α*. Prophylaxis with GMP also diminished serum anti-OVA IgE and IgG1, and histamine levels. GMP treatment markedly decreased eosinophil infiltration, mast cell and goblet cell hyperplasia, total *IgE* expression in intestine, and prevented histological changes in villi, crypts and internal muscularis layer. The treatment effectively suppressed *IL-5*, *IL-13* and *GATA3* expression and skewed the intestinal cytokine profile toward type 1 and regulatory. These results suggest that GMP may protect against FA through down-regulating the type 2 inflammatory response.

## 1. Introduction

Food allergy (FA) is an adverse immune reaction to food proteins that is becoming a growing clinical problem. The World Allergy Organization has declared that 2.5% of the general population suffers from some type of FA [1]. Mainly, the child population is affected, causing a strong economic impact in families and in the health care systems. A survey about FA carried out in the US reveals a prevalence of 8% in children, with an overall estimated cost of 24.8 trillion dollars annually [2,3]. FA can be grouped in immunoglobulin (Ig)E-mediated, non-IgE-mediated or the condition combining IgE and non-IgE mechanisms [4]. However, IgE-mediated allergy is associated with an increased risk of severe, even fatal, reactions [5]; accordingly, this type of FA is the most studied in order to find effective methods of treatment. The clinical manifestations include a wide spectrum of cutaneous, gastrointestinal (GI), respiratory or cardiovascular symptoms [4,5], without a hierarchical order nor dependency on each other. Skin and GI tract are most commonly affected sites after a food challenge, although it depends on the allergen [6]. In childhood, egg is one of the most common food related to FA. The ovalbumin (OVA) is found among the most allergenic and abundant proteins in the white egg [4,7,8,9]. GI symptoms are the most frequently presented after egg consumption [6].

In IgE-mediated FA, the immune response to food proteins that pass through the disrupted epithelial barrier is developed within a tissue environment governed by inflammatory cytokines that activates dendritic cells to in turn activate T-cells into a T helper 2 (Th2) phenotype, which then prime the production of allergen specific IgE by B lymphocytes. Afterwards, IgE is bound to the high affinity receptor FcεRI on mast cells and the individual becomes allergen-sensitized [10]. The subsequent intake of the food allergen triggers the activation of mast cells, inducing the release of preformed and *de novo* produced vasoactive and inflammatory mediators, that causes the immediate manifestations of the allergic response [4,11]. While the connective tissue mast cell degranulation is related with systemic manifestations and anaphylactic shock, mucosal mast cell degranulation is associated with GI symptoms, such as diarrhea [12]. The symptoms in this FA stage are essentially functional, acute and rapidly reversible [13]. Afterwards, allergic inflammation in gut is maintained by continuous high levels of type 2 cytokines such as interleukin (IL)-4, IL-5 and IL-13, and by the recruitment of other inflammatory cells, such as eosinophils, neutrophils, monocytes, macrophages, and T lymphocytes. Cytokines and toxic compounds released by these cells cause the late-phase reaction of FA, in which inflammation becomes chronic and initiates the damage within the tissue [11]. At this stage, the symptoms are more slowly reversed [13].

The use of FA animal models is essential to analyze the immunological mechanisms involved in the onset and development of the disease and to establish new treatment strategies [14]. The animal models are adequate as long as they have a GI system similar to the one of humans, produce IgE antibodies and manifest similar allergic reactions in response to food allergens. Among rodent models, Brown Norway and Wistar rats meet these conditions [15]. Although Brown Norway rats have been traditionally claimed as the most suitable model to study FA, Wistar rats have recently been demonstrated to increase IgE levels after oral immunization with OVA and to develop the key clinical manifestations of FA to peanut allergens [16,17,18]. Thus, Wistar rats have the potential to be used as an experimental model of IgE-mediated FA with GI manifestations in response to OVA.

The study of natural bioactive compounds as potential therapies in FA has attracted researcher attention due to the increased prevalence and costs of FA and the absence of a definitive treatment [4,18,19]. Several recent reports have shown that some bioactive peptides derived from milk may exert beneficial effects on preventing or treating human chronic inflammatory diseases [20]. Glycomacropeptide (GMP) is a bioactive peptide derived from the hydrolysis of milk κ-casein by chymosin during cheese manufacturing or by pepsin during the digestion process [21]. This highly glycosylated 64-amino acid peptide presents an average molecular weight of 7.5 kDa [22]. It is considered safe for human use and is not immunogenic regardless of the administration route [23,24]. GMP is free from phenylalanine residues, which allows it to be used as a protein-based ingredient in the formulation of food products for the nutrition of phenylketonuria patients [25]. Many different biological activities have been attributed to GMP [26]. Among them, the immune-modulatory and anti-inflammatory effects of orally administered GMP. Indeed, mice fed with a diet supplemented with GMP show decreased level of serum IgG antibodies against dietary OVA or intraperitoneally injected β-lactoglobulin [27]. In addition, GMP intake also reduces the proliferative response of splenocytes induced by mitogens in the rat [28]; a widely validated property of GMP by in vitro assays [29,30,31]. When GMP is orally and prophylactically administered to rats, it reduces the type 2 inflammatory response and prevents lung and skin damage in experimental models of asthma and atopic dermatitis [32,33]. In both studies, GMP increases *IL-10* expression on affected tissues. Besides, it has been shown that GMP reduces intestinal inflammatory cytokine levels and oxidative stress in experimental models of colitis, ileitis and nonsteroidal anti-inflammatory drug-induced (NSAID) enteropathy [34,35,36,37]. These mentioned bioactive properties suggest that the use of GMP may regulate the immune response and protect against the intestinal inflammation and damage associated to FA.

The present study was designed to evaluate whether oral administration of GMP can prevent the development of FA in the rat. First, we characterized a rat model of FA with GI signs by systemic sensitization with OVA followed by repeated oral challenges with the allergen. We further assess the effect of GMP on antibody production, clinical manifestations, intestinal inflammation, and jejunum morphological alterations associated to FA. Finally, we examined whether GMP treatment is able to modulate the type 2 inflammatory response that underlies FA development.

## 2. Materials and Methods

### 2.1. Animals

Male Wistar rats (8 weeks old, 100–120 g) were obtained from the Laboratory Animal Service of the Autonomous University of Aguascalientes. Animals were maintained under controlled conditions of temperature (22–24 °C) and illumination (12 h light-dark cycle) and provided with an egg-protein free rodent chow diet (Nutricubo, Purina, USA) since weaning, and tap water *ad libitum*. The parent rats also followed a diet free of egg proteins. Rats were housed in the animal facility at the Basic Science Center of the University in metallic cages (5 animals/cage) with adequate sawdust substrate and under the same conventional conditions, and were acclimated during 7 days prior to the beginning of the FA protocol. Experimental protocols in this study were approved by the Institutional Ethical Committee for the Use of Animals in Research (approval date: 02 April 2018) and complied with Animal Welfare Mexican Law (NOM-062-ZOO-1999) and National Institute of Health Guide for the Care and Use of Laboratory Animals.

### 2.2. Protocols for Induction of Experimental Food Allergy

**Figure 1:**
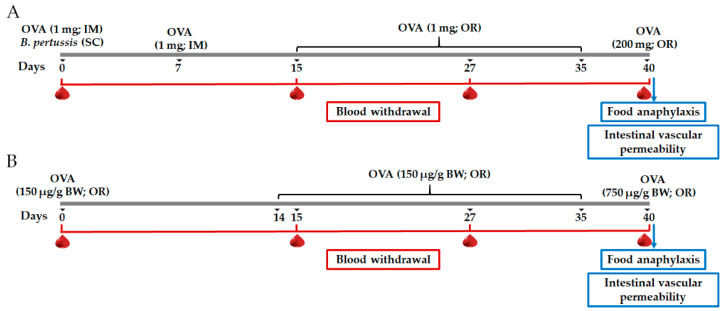
Schematic diagram of the food allergy (FA) experimental protocols. (**A**) FA protocol developed by intramuscular (IM) sensitization and oral (OR) challenge with ovalbumin (OVA); (**B**) FA protocol developed by OR sensitization and OR challenge with OVA. SC, subcutaneous; BW, body weight.

For the characterization of the FA model, rats (*n* = 10) were randomly assigned to the following groups: sham control of intramuscular sensitization and oral challenge (SH-IM), FA-induced by intramuscular sensitization and oral challenge (FA-IM), sham control of oral sensitization and oral challenge (SH-O), FA-induced through oral sensitization and oral challenge (FA-O). OVA (Sigma Aldrich, St. Louis, MO, USA) was used as allergen. For intramuscular sensitization (Figure 1A), at day 0 the FA-IM animals received intramuscularly 1 mg of OVA adsorbed in 7.8 mg of aluminum hydroxide (Imject Alum; Thermo Scientific, Waltham, MA, USA) dissolved in saline solution (1 mL), and subcutaneously 4 IU of *Bordetella pertussis* (Dipertix; Biofarma, Bandung, Indonesia). At day 7, animals received an intramuscular booster of the allergen [28]. Later, sensitized animals were orally challenged with 1 mg OVA prepared in 0.2 M NaHCO_3_ (1 mL; Sigma Aldrich, St. Louis, MO, USA) from day 15 to 35 in 5-day periods per week (3-day rest) [38]. For oral sensitization (Figure 1B), at day 0 the FA-O animals received orally 150 μg of OVA/g body weight dissolved in 0.2 M NaHCO_3_ (1 mL). Afterwards, sensitized animals were challenged with the same allergen dose daily from day 14 to 35 [16]. Animals from the SH-IM and SH-O groups carried out the corresponding protocol without OVA administration. At day 40 and after a 24-h fasting period, rats of SH-IM and FA-IM groups received orally a final challenge of 200 mg of OVA and animals of SH-O and FA-O groups of 750 μg of OVA/g body weight, in both cases dissolved in 0.2 M NaHCO_3_ (2 mL). All oral administrations were made using an esophageal feeder.

Six rats per group were used to collect blood and to induce food anaphylaxis. Blood was obtained at day 0 (before OVA sensitization), 15, 27 and 40 by puncture of the tail vein. After clotting, samples were centrifuged at 735× *g* for 15 min and the sera were aliquoted and stored at −80 °C until use. Four rats per group were used to assay intestinal vascular permeability.

### 2.3. Experimental Design and Sample Collection

**Figure 2:**
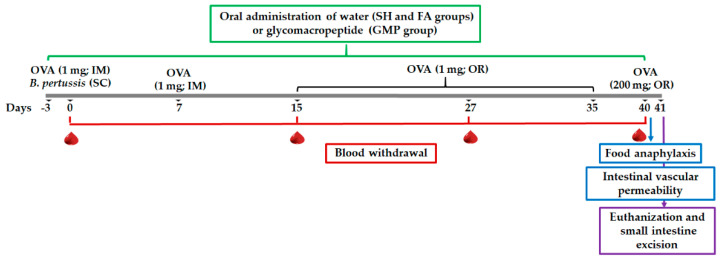
Schematic diagram of experimental setup and glycomacropeptide (GMP) administration. FA protocol was developed in rats by intramuscular (IM) sensitization and oral (OR) challenge with ovalbumin (OVA). SC, subcutaneous; SH, sham control group; FA, FA-induced group; GMP, FA-induced and GMP-treated group.

Rats were randomly assigned into three different groups (*n* = 24): sham control (SH), FA-induced (FA), and FA-induced and GMP-treated (GMP). The protocol of intramuscular sensitization and oral challenge with OVA was carried out in animals. Rats from GMP group received daily and orally 500 mg/kg/day of GMP (Lacprodan cGMP-10 gifted by Arla Food Ingredients Group P/S, Viby, Denmark) dissolved in sterile tap water; while for the SH and FA groups, animals received 1 mL of sterile tap water. Treatment was administered using an esophageal catheter from three days prior to the first immunization until day 40, and 4 h after OVA or NaHCO_3_ administration (Figure 2). The dose of GMP used in this study was established based on previous therapeutic studies in rats [32,33,34,35,36].

In each group, ten rats were used to induce food anaphylaxis, five animals to assess intestinal vascular permeability, and an additional nine rats were used for blood collection throughout the protocol. Blood was obtained and processed as indicated above. Twenty-four hours after the allergen final challenge, five randomly chosen rats per group were euthanized by intraperitoneal sodium pentobarbital overdose (≥100 mg/kg; Pisa Agropecuaria, Atitalaquia, Mexico) and the small intestines were excised and divided in two equal parts. A 3-cm segment of the proximal jejunum was dissected and fixed in neutral formalin (Sigma-Aldrich, St. Louis, MO, USA) for histological analysis. The next 1-cm segment was collected and stored at −80 °C in RNAlater (Ambion, Austin, TX, USA) for the mRNA analysis.

### 2.4. Determination of Allergen-Specific IgE and IgG1 in Serum

Levels of OVA-specific IgE in serum were analyzed by the passive cutaneous anaphylactic reaction, as previously described [28]. Briefly, sera from SH, FA and GMP rats were individually analyzed by intradermal injection of two-fold dilutions (from 1:2 to 1:128) in the shaved dorsal skin of normal Wistar rats (14 weeks old, 300–350 g). One day later, saline and histamine (0.04 μg/μL, Sigma-Aldrich, St. Louis, MO, USA) solutions were injected as negative and positive controls, respectively. Immediately, an intravenously (jugular vein) injection of 2 mg OVA and 34 mg/kg Evans blue (Sigma-Aldrich, St. Louis, MO, USA) in 3% saline solution was performed. After 30 min, rats were euthanized and the dorsal skin was inverted to measure the diameter of the blue spots generated at the injection sites of each diluted serum, histamine or saline solutions using a digital Vernier caliper. The IgE anti-OVA antibody titer for each serum was expressed as the highest dilution causing a lesion of more than 5 mm in diameter and was represented as the inverse of the titer [39].

The allergen specific IgG1 level in the serum was evaluated by an indirect semi-quantitative ELISA system using a previously described method with slight modifications [16]. Briefly, 96-well plates (Thermo Scientific, Waltham, MA, USA) were coated with 10 µg/mL OVA in 50 mM carbonate buffer overnight in constant shaking at 4 °C. After washing the plates with PBS containing 0.05% Tween-20 (PBST; Sigma-Aldrich, St. Louis, MO, USA), wells were blocked with 50 mM carbonate buffer with 1% bovine serum albumin (BSA; Equitech-Bio, Kerrville, TX, USA) 30 min at room temperature (RT). Then, the plates were incubated with serum samples diluted 1:50 with PBST containing 1% BSA during 1 h and later with mouse biotinylated monoclonal antibody anti-rat IgG1 (1:2000, ABCAM, Cambridge, United Kingdom) 2 h at 37 °C. After three washings with PBST, HRP-streptavidin (1:60,000, ABCAM, Cambridge, United Kingdom) was added to the plates for 1 h at 37 °C. Wells were washed three times with PBST, developed with *O*-phenylenediamine (Sigma Aldrich, St. Louis, MO, USA) for 20 min at RT, and reaction was stopped by addition of 4 N H_2_SO_4_ (Sigma Aldrich, St. Louis, MO, USA). Optical density (OD) was read at 490 nm in an iMark plate spectrophotometer (Bio-Rad, Hercules, CA, USA). The IgG1 level of experimental samples was determined in relation to those of non-sensitized rats by dividing the net OD of each sample by the positive index (PI), that was calculated as OD average +2 standard deviation of 10 serum samples from non-sensitized rats.

The global production of allergen-specific Igs over time was evaluated by calculating the area under the curve (AUC).

### 2.5. Quantification of Serum Histamine and Rat Mast Cell Protease-2

Level of histamine and rat mast cell protease (rMCP)-2 were quantified in serum obtained 30 min after allergen challenge at days 27 and 40 [16], or 120 min after allergen challenge at day 40 [38], respectively. Commercial ELISA kits were used following the manufacturer’s instructions. Histamine was measured by a competitive solid phase ELISA (MBS732202, MyBiosource, San Diego, CA, USA) and rMCP-2 by an ELISA sandwich assay (OKEH02490, Aviva Systems Biology, San Diego, CA, USA). After stopping the reaction, OD was measured at 450 nm in an iMark plate spectrophotometer.

### 2.6. Food Anaphylaxis

The severity of anaphylaxis was evaluated by scoring the clinical signs every 10 min during the first hour and at 1.5, 2 and 24 h after dosing the final allergen challenge at day 40. Clinical signs were graded by the following score scale: 0, no signs; 1, scratching and rubbing around the nose and head; 2, puffiness around the eyes and mouth, diarrhea, pilar erecti, reduced activity, and/or decreased activity with increased respiratory rate; 3, wheezing, labored respiration, and cyanosis around the mouth and the tail; 4, no activity after prodding or tremor and convulsion; 5, death [40]. For the characterization of the FA model, rectal temperature was measured using a digital thermometer (CT-513W, Citizen, Tokyo, Japan) at the same times as the clinical signs. The features of diarrhea in treated animals was analyzed using the following score to grade changes in stool appearance: 0, no changes; 1, soft but well formed; 2, soft, non-formed; 3, one episode of liquid diarrhea; 4, at least two episodes of liquid diarrhea [41].

### 2.7. Intestinal Edema

Vascular permeability changes were evaluated by Evans blue dye extravasation. Animals were intravenously injected (at the jugular) with 20 mg/kg Evans blue dye in 1.5% saline solution at day 40 [42]. Five min later, the animals were orally challenged with the allergen (final challenge) using an esophageal feeder. After 30 min, rats were euthanized and the abdomen was opened to dissect 5-cm segments of the duodenum, proximal jejunum and ileum. Intestinal tissues were washed with PBS (pH 7.4) to eliminate the feces and then four times with a 6 mmol/L acetylcysteine solution (Sigma Aldrich, St. Louis, MO, USA) to decrease mucin viscosity. Each segment was immersed in formamide (Sigma Aldrich, St. Louis, MO, USA) for 24 h at 50 °C to extract the Evans blue dye. Then, samples were centrifuged at 100× *g* for 10 min. The amount of extravasated dye was determined by measuring the absorbance at 620 nm, using a standard curve (0.1 to 30 μg/mL Evans blue).

### 2.8. Quantification of Gene Expression in Small Intestine

Total intestinal RNA was isolated from the 1-cm segment of proximal jejunum using the GeneJET RNA purification kit (Thermo Scientific, Waltham, MA, USA). Then, 1 µg of purified RNA was treated with RQ1 RNase-Free DNase (Promega, Madison, WI, USA). Complementary DNA was synthetized with a Maxima First Strand cDNA Synthesis Kit (Thermo Scientific, Waltham, MA, USA) in a 2720 Thermal Cycler (Applied Biosystems, Foster City, CA, USA). Quantitative real time PCR was established with the Maxima SYBR Green/ROX qPCR Master Mix (Thermo Scientific, Waltham, MA, USA) in a StepOne Real-Time PCR System (Applied Biosystems, Foster City, CA, USA). Primers used for the quantification of mRNA expression are listed in Table 1. The relative quantification was determined with the 2^−ΔΔCt^ method, normalizing with the β-actin as a housekeeping gene [43]. The ratio of the transcription factors was calculated within each animal and means were compared.

### 2.9. Histological Analysis

Fixed intestinal tissue was embedded in paraffin, sliced in 5-µm sections and mounted on adhesive slides (Klinipath, Duiven, The Netherlands). After deparaffination and rehydration, slices were stained with hematoxylin (Diapath, Martinengo, Italy) and eosin (RAL diagnostic, Martillac, France) contrasted with saffron (RAL diagnostic, Martillac, France) (HES) for intestinal histomorphometric examination and eosinophil identification; and Schiff’s periodic acid (PAS; Diapath, Martinengo, Italy) to identify goblet cells. Slides were scanned with the Pannoramic SCAN II (@BRIDGe platform, INRAE, Jouy en Josas, France). The morphometric analysis was made with the Case Viewer 3D Histech software version 2.3 (3DHistech, Budapest, Hungary) of the same scanner, in 3 slices per animal and in a blinded fashion. Intestinal histomorphometric parameters were evaluated by measuring villus height, crypt depth and internal *muscularis* thickness. Eight villi and 10 crypts randomly chosen were analyzed per slice. Villus height was defined as the distance between the villus apex and villus-crypt junction, and the crypt depth was measured as the distance from the villus-crypt junction to the crypt base. In addition, ten random thickness measurements of the internal *muscularis* layer were performed per slice. Eosinophils were counted in 20 randomly chosen fields of 5000-µm^2^ surface and goblet cells in one randomly chosen field of 1-mm^2^ surface per slice and expressed as the number of cells per area.

### 2.10. Statistical Analysis

Statistical analysis was performed using the GraphPad Prism v.8.01 software (GraphPad, San Diego, CA, USA). Comparisons among groups were made with t-Student test or one-way ANOVA with *post hoc* Bonferroni’s multiple comparisons test; except for IgE and IgG1 outcomes in which two-way ANOVA with a *post hoc* Holm-Sidak´s multiple comparisons analysis was made and the AUC was obtained. Data are expressed as the mean ± standard error of the mean (S.E.M). Anaphylactic and diarrhea scores were analyzed by Mann-Whitney test or Kruskall-Wallis with *post hoc* Dunn´s test, and data are expressed as median. Differences were considered statistically significant when *p* < 0.05.

## 3. Results

### 3.1. Characterization of IgE-Mediated Food Allergy Evoked by Systemic or Oral Sensitization with Allergen

**Figure 3:**
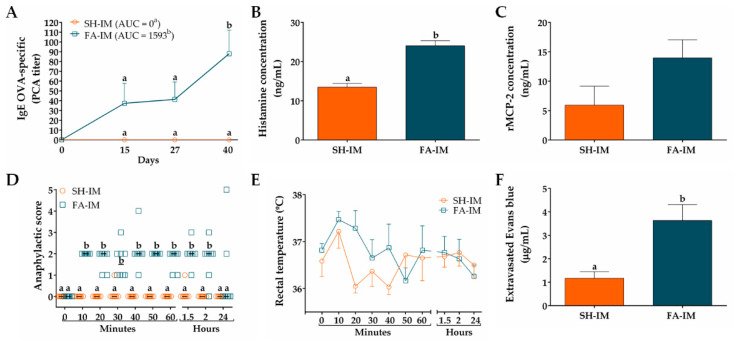
Characteristics of IgE-mediated food allergy (FA) induced by systemic allergen sensitization. (**A**) IgE OVA-specific titers in serum at different times measured by passive cutaneous anaphylaxis (PCA) reaction and represented as the inverse of the titer (*n* = 6); the values of area under the curve (AUC) were evaluated. Concentration of (**B**) histamine and (**C**) rat mast cell protease (rMCP)-2 in serum 30 and 120 min after OVA challenge at days 27 and 40, respectively (*n* = 3). Food anaphylaxis evaluated by (**D**) signs score and (**E**) rectal temperature of animals after the final allergen challenge at day 40 (*n* = 6). (**F**) Intestinal edema of rats after the final allergen challenge analyzed by intestinal extravasated Evans blue (*n* = 4, in triplicate). SH-IM, sham control of intramuscular sensitization and oral challenge; FA-IM, FA-induced by intramuscular sensitization and oral challenge. Different letters (a,b) indicate statistical difference.

We first characterized the IgE-mediated FA model with GI manifestations in Wistar rats induced by initial systemic allergen-sensitization using adjuvants (FA-IM group) or by initial oral allergen-sensitization without adjuvants (FA-O group). The results of FA model raised by systemic allergen-sensitization are shown in Figure 3. The OVA-specific IgE titer of sera obtained prior to immunization (day 0), as well as that of the sham animals (SH-IM group) at different times throughout the whole protocol, was zero. However, the sera of the FA-IM animals showed an increase in anti-OVA IgE titer at day 15 (37.33 ± 20.39), that remained constant at day 27 (41.33 ± 17.82), and significantly peaked at day 40 (88 ± 24; *p* < 0.05; Figure 3A). Values of AUC of IgE OVA-specific titers were higher in FA-IM group than in SH-IM group (1593 vs. 0; *p* < 0.05). Serum histamine level increased 78% at day 27 in FA-IM animals (24.02 ± 1.32 ng/mL) compared to SH-IM animals (13.46 ± 0.98 ng/mL, *p* < 0.01; Figure 3B). When histamine was measured at day 40, no change was observed between both groups (12.13 ± 0.16 ng/mL FA-IM vs. 10.14 ± 0.82 ng/mL SH-IM). Serum level of rMCP-2 augmented 135% in FA-IM rats in comparison to SH-IM animals, although this rise was not significant (13.95 ± 3.08 ng/mL vs. 5.92 ± 3.23 ng/mL; Figure 3C). To evaluate whether food anaphylaxis developed in the experimental model of FA after intake of allergen, and whether animals presented systemic or GI manifestations, clinical signs and changes in rectal temperature were scored after the final oral challenge with OVA. As shown in Figure 3D, the animals from the SH-IM group did not show clear signs of food anaphylaxis during 24 h after allergen intake. Conversely, the animals of the FA-IM group, at 10 min after allergen challenge, displayed intense scratching and pilar erecti, accomplishing a median of score of 2 (*p* < 0.005). These clinical signs were followed by continuous events of diarrhea after the first 40 min and were constantly manifested during two hours. The rectal temperature was also recorded in rats over time, showing no significant changes among groups and ranging from a maximum of 37.5 °C to a minimum of 36.0 °C (Figure 3E). Finally, intestinal edema after oral allergen challenge was determined by quantifying extravasated Evans blue in intestinal tissue (Figure 3F). FA-IM animals showed 3.1-fold higher intestinal dye extravasation than SH-IM animals (3.63 ± 0.68 µg/mL vs. 1.16 ± 0.27 µg/mL; *p* < 0.01).

**Figure 4:**
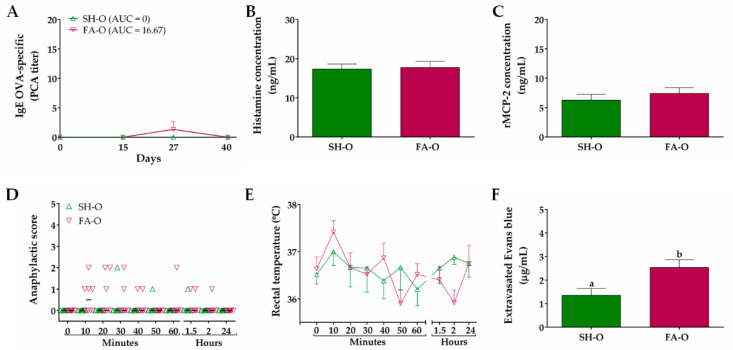
Characteristics of IgE-mediated food allergy (FA) induced by oral allergen sensitization. (**A**) IgE OVA-specific titers in serum at different times measured by passive cutaneous anaphylaxis (PCA) reaction and represented as the inverse of the titer (*n* = 6); the values of area under the curve (AUC) were evaluated. Concentration of (**B**) histamine and (**C**) rat mast cell protease (rMCP)-2 in serum 30 and 120 min after OVA challenge at days 27 and 40, respectively (*n* = 3). Food anaphylaxis evaluated by (**D**) signs score and (**E**) rectal temperature of animals after the final allergen challenge at day 40 (*n* = 6). (**F**) Intestinal edema of rats after the final allergen challenge analyzed by intestinal extravasated Evans blue (*n* = 4, in triplicate). SH-O, sham control of oral sensitization and oral challenge; FA-O, FA-induced by oral sensitization and oral challenge. Different letters (a,b) indicate statistical difference.

In relation to FA model established by oral allergen-sensitization, the OVA-specific IgE titer of sera obtained prior to immunization (day 0), as well as that of the SH-O group at different times throughout the whole protocol, was zero (Figure 4A). In FA-O animals, IgE titer only showed a slight increase at day 27 (1.33 ± 1.33), with an AUC value of 16.67 (not significant difference vs. SH-O). The serum concentration of histamine and rMCP-2 was not modified in animals from the FA-O group as compared to SH-O animals (Figure 4B,C). The animals from the SH-O group did not show clear signs of food anaphylaxis during 24 h after allergen intake and FA-O animals only exhibited an occasional scratching and rubbing around the nose and head, with a median of score close to 0 from 10 min to 24 h after allergen challenge (not significant difference vs. SH-O; Figure 4D). The rectal temperature recorded in rats over time showed no significant changes among groups (Figure 4E). Strikingly, intestinal edema in FA-O animals was 1.9-fold higher than in SH-O animals (2.53 ± 0.33 µg/mL vs. 1.35 ± 0.28 µg/mL, *p* < 0.05; Figure 4F).

Thus, initial systemic allergen-sensitization using adjuvants is needed in Wistar rats to induce an IgE-mediated FA model that develops food anaphylaxis with GI manifestations and shows intestinal inflammation in response to oral allergen challenge.

### 3.2. GMP Administration Modulates Immunoglobulin Production in Response to Allergen Sensitization

**Figure 5:**
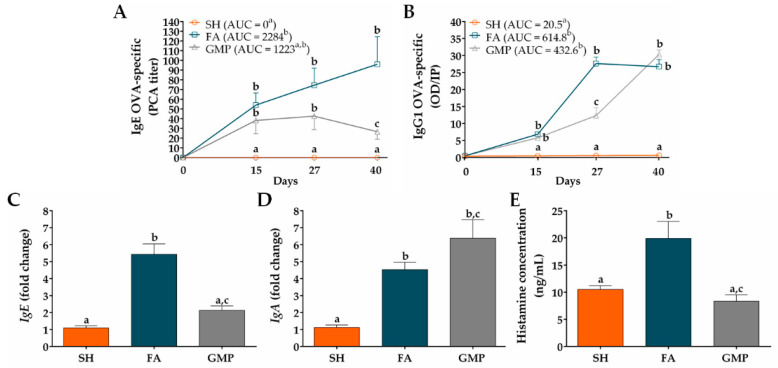
Effect of treatment with glycomacropeptide (GMP) on immunoglobulin and histamine levels in rats with food allergy (FA). (**A**) Ovalbumin (OVA)-specific IgE titers were measured in serum at different times by passive cutaneous anaphylaxis (PCA) reaction and represented as the inverse of the titer (*n* = 9); the values of area under the curve (AUC) were evaluated. (**B**) OVA-specific IgG1 levels were measured in serum at different times by ELISA and represented as optical density (OD) of sample in relation to positive index (PI) (*n* = 9); the values of AUC were evaluated. Intestinal expression level of total (**C**) *IgE* and (**D**) *IgA* relative to β-actin was analyzed by quantitative PCR and expressed as fold change (*n* = 4). (**E**) Histamine concentration was measured by ELISA in serum samples obtained 30 min after allergen challenge at day 27 (*n* = 9). SH, sham control; FA, FA-induced; GMP, FA-induced and GMP-treated animals. Different letters (a,b,c) indicate statistical difference.

In order to analyze whether GMP treatment may modulate the sensitization status of animals, OVA-specific IgE serum levels were measured before and after allergen sensitization and oral challenges. As shown in Figure 5A, sham animals did not present allergen-specific IgE in serum. OVA-immunization triggered the production of anti-OVA IgE antibody in FA animals, with an average titer of 54 ± 12.44 at day 15 (*p* < 0.05). The oral administration of the allergen boosted the rise of this immunoglobulin that peaked on day 40 (96 ± 28.36; *p* < 0.05). Rats with FA but treated with GMP (ie. GMP group) also produced allergen-specific IgE, showing an antibody average titer of 38.22 ± 13.58 at day 15 (*p* < 0.05), but this value remained constant during allergen oral challenges (42.67 ± 13.79, at day 27; *p* < 0.05), and presented a slight reduction at day 40 (26.67 ± 7.86; *p* < 0.05). At this time, anti-OVA IgE level was 72% less in GMP-treated animals than in FA group (*p* < 0.05). When values of AUC were analyzed, outcomes showed that IgE titer significantly increased in FA group compared to SH animals (*p* < 0.05), and although GMP treatment half reduced this AUC value, the effect was not significant. Another immunoglobulin related to the allergic response is IgG1 [38]. Allergen-specific IgG1 level in serum of sham animals was zero (Figure 5B). Both FA and GMP animals synthesized anti-OVA IgG1, showing similar levels at day 15 (6.84 ± 0.59 and 5.76 ± 1.06; *p* < 0.01 vs. SH). However, after allergen oral challenges, this immunoglobulin increased rapidly in FA animals to reached its maximum value at day 27 (27.62 ± 1.91; *p* < 0.0001), while in animals treated with GMP the concentration was 55% less (12.31 ± 2.27; *p* < 0.001 vs. FA and SH). At day 40, the level of anti-OVA IgG1 was the same in both FA and GMP animals (26.69 ± 2.12 and 30.30 ± 1.29; *p* < 0.0001 vs. SH). The AUC value of IgG1 titer was similar between FA and GMP groups, and in both groups it was significantly higher than that of SH animals (*p* < 0.0001). We also investigated the effect of GMP treatment on mRNA expression level of total *IgE* and *IgA* in the rat intestinal tissue at the end of the study. We found that the oral challenge with allergen triggered *IgE* and *IgA* gene expression in small intestine of FA animals, with an approximate 5- and 4-fold higher expression (*p* < 0.0001 and *p* < 0.05, respectively) than in SH animals (Figure 5C,D). Treatment with GMP significantly reduced *IgE* gene expression by 61% (*p* < 0.001), whereas *IgA* mRNA level was slightly but not significantly increased. Finally, we studied the serum histamine concentration after the allergen challenge at day 27 (Figure 5E). The histamine amount was 19.91 ± 3.12 ng/mL in FA animals in comparison to 10.48 ± 0.71 ng/mL in sham animals, thus showing for this mast cell mediator a 1.9-fold increase in serum as a consequence of IgE production and mast cell activation by allergen (*p* < 0.01). In accordance with OVA-specific IgE levels, GMP treatment decreased serum histamine values of allergic animals (8.31 ± 1.21 ng/mL; *p* < 0.01).

### 3.3. Oral Administration of GMP Reduces the Severity of Gastrointestinal Manifestations after Allergen Intake

**Figure 6:**
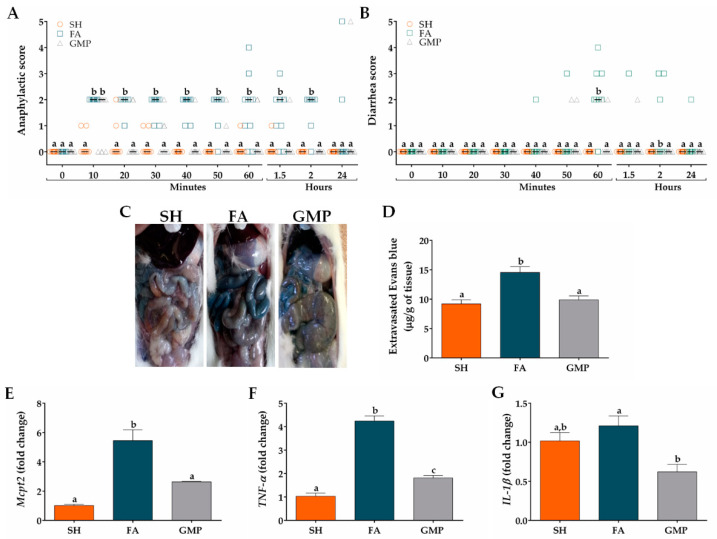
Effect of treatment with glycomacropeptide (GMP) on food anaphylaxis severity and intestinal inflammation. (**A**) Food anaphylaxis severity was evaluated by scoring clinical signs of animals from 10 min to 24 h after last allergen oral challenge (*n* = 10). (**B**) Diarrhea severity was scored in animals from 10 min to 24 h after last allergen oral challenge (*n* = 10). (**C**) Representative images of small intestine of animals intravenously injected with Evans blue dye and later orally challenged with allergen. (**D**) Graph of concentration of extravasated Evan blue within intestine and expressed as µg of dye/g of tissue (*n* = 5, in triplicate). Intestinal expression level of (**E**) *Mcpt2*, (**F**) *TNF-α* and (**G**) *IL-1β* relative to *β-acti*n was analyzed by quantitative PCR and expressed as fold change (*n* = 4). SH, sham control; FA, FA-induced; GMP, FA-induced and GMP-treated animals. Different letters (a,b,c) indicate statistical difference.

In order to investigate whether GMP administration impacts the severity of food anaphylaxis, clinical signs were evaluated in FA animals treated or not with GMP. Animals of FA group initiated with signs of food anaphylaxis from 10 min to 2 h after allergen challenge, with a median of score of 2 (Figure 6A). The main clinical signs manifested by animals, as mentioned before, were intense scratching, pilar erecti and diarrhea. The treatment with GMP significantly reduced the severity of food anaphylaxis, as animals showed an anaphylactic score with a median of 0 from 20 min to 2 h after allergen challenge (*p* < 0.05). The main sign manifested by GMP animals was a slight scratching and it disappeared entirely at 2 h. Twenty-four hours after allergen challenge, there was not a significant difference in the anaphylactic score between animals from GMP and FA group. As the main GI sign in animals was diarrhea, we evaluated diarrhea severity from 10 min after allergen challenge to 2 h. As shown in Figure 6B, none of the SH animals defecated after the challenge or their stools had normal consistency. However, most of the FA animals started with diarrheal episodes from 40 min after the allergen challenge, with a maximum median of score of 2 at 60 min (*p* < 0.001). At this time, 20% of animals defecated non-formed soft stools, 30% presented one episode of liquid diarrhea and 10% had more than two episodes of liquid diarrhea. GMP treatment significantly attenuates diarrhea severity, as animals presented a median of score of 0 (*p* < 0.01) at 60 min, and only 18% of animals showed soft consistency in their stools. To assess the intestinal edema caused by food anaphylaxis, animals were intravenously injected with Evans blue dye just before the last oral challenge (Figure 6C). Thirty min later and due to vascular leak, Evans blue concentration in intestinal tissue of FA animals was 14.55 ± 0.97 μg/g, 1.5 times higher than sham animals (9.19 ± 0.71 μg/g of tissue, *p* < 0.0001; Figure 6D). GMP treatment abolished the intestinal edema induced by oral allergen challenge (9.88 ± 0.66 μg of dye/g of tissue; *p* < 0.001). Since mucosal mast cells have been implicated in the allergic diarrhea [12], which was the main GI manifestation of FA animals, we proceeded to analyze the effect of GMP treatment on the number of mucosal mast cells in the inflamed intestine. For this, we performed qPCR assays to analyze *Mcpt2* mRNA expression (gene encoding rMCP-2). Results showed a clear increase in mRNA expression of *Mcpt2* in FA animals when compared to control animals (*p* < 0.001; Figure 6E), which decreased by 52% when animals were GMP-treated (*p* < 0.01). Finally, we evaluated the expression level of *TNF-α* and *IL-1β*, two mast cell-derived cytokines related with allergic inflammation [44,45]. Allergen challenge clearly increased the level of *TNF-α* in intestinal tissue of FA animals as compared to sham group (*p* < 0.001; Figure 6F). When animals were GMP-treated *TNF-α* level was 57% reduced (*p* < 0.001). Nevertheless, the gene expression of *IL-1β* was not affected by the OVA challenge, although animals that received GMP decreased by 49% *IL-1β* expression (*p* < 0.05; Figure 6G).

### 3.4. Oral Administration of GMP Improves the Pathophysiology of the Intestinal Mucosa in Food Allergy Animals

**Figure 7:**
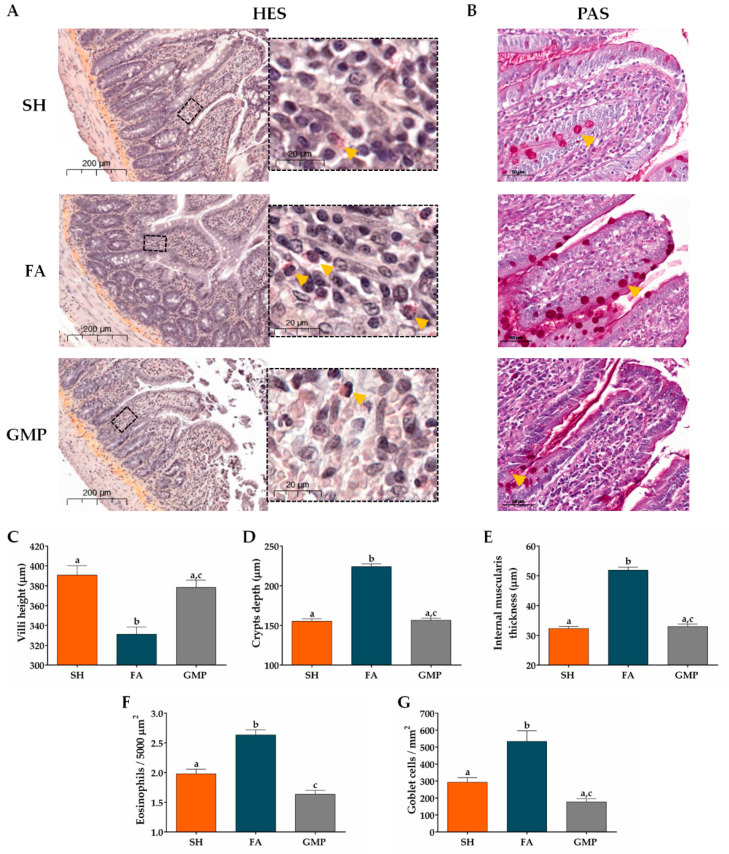
Effect of treatment with glycomacropeptide (GMP) on the pathophysiology of the intestinal mucosa in rats with food allergy (FA). Sections of jejunum were stained with (**A**) hematoxilin-eosin-saffron (HES) to analyze morphological alterations of mucosa and to identify eosinophils (yellow arrows), or with (**B**) Schiff’s periodic acid (PAS) to identify goblet cells (yellow arrows). Morphometric analysis was made with the Case Viewer 3D Histech software. The (**C**) villus height (μm), (**D**) crypt depth (μm), and (**E**) internal *muscularis* layer thickness (μm) were measured in samples of small intestine obtained from experimental animals. Quantitative analysis of (**F**) eosinophils and (**G**) goblet cells in intestinal tissue was developed in fields of 5000-µm^2^ and 1-mm^2^ surface, respectively. SH, sham control; FA, FA-induced; GMP, FA-induced and GMP-treated animals. Different letters (a,b,c) indicate statistical difference.

We evaluated the influence of GMP treatment on jejunum morphological alterations associated with FA using histochemical examination. As shown in Figure 7A, results from HES stain showed irregular shapes of the villi, hyperplasia in the crypts, thickness of internal *muscularis* layer and eosinophilic infiltrates in animals from FA group as compared to SH animals; and PAS stain (Figure 7B) revealed the hyperplasia of goblet cells in the intestinal epithelium of FA rats compared to SH group. These results demonstrate typical pathological features of allergy to food in intestine of FA animals [46]. Interestingly, histological observation also showed that treatment with GMP attenuated these morphological alterations. To quantify the intestinal alterations, villus height, crypt depth and the thickness of the circular *muscularis* were measured. Morphometry revealed that villus height was 15% less in FA that in sham animals (331.00 ± 7.26 µm vs. 390.58 ± 9.61 µm, *p* < 0.001; Figure 7C), while crypt depth and internal thickness was 44% and 60% greater (223.96 ± 3.64 µm vs. 155.44 ± 2.73 µm and 51.83 ± 1.06 µm vs. 32.31 ± 0.72 µm, *p* < 0.001; Figure 7D,E). Strikingly, administration of GMP prevented the intestinal morphological changes induced by the FA, restoring values of villi height (378.35 ± 7.17 µm; *p* < 0.001), crypts depth (156.54 ± 2.79 µm; *p* < 0.001) and *muscularis* internal thickness (32.97 ± 0.80 µm; *p* < 0.001). As FA is characterized by a marked eosinophilia in intestinal tissue [47], we also evaluated eosinophil infiltration in gut lamina propria. Eosinophil amount in FA animals was 1.3-fold higher than in sham animals (2.63 ± 0.08 cells/5000 µm^2^ vs. 1.97 ± 0.08 cells/5000 µm^2^, *p* < 0.001; Figure 7F). GMP treatment avoided the eosinophilia in lamina propria of animals with FA (1.63 ± 0.06 cells/5000 µm^2^; *p* < 0.001). Finally, morphometric assessment showed that the number of goblet cells in the jejunum of FA animals was 1.8-fold higher compared to sham animals (533.40 ± 62.50 cells/mm^2^ vs. 292.73 ± 27.58 cells/mm^2^, *p* < 0.001; Figure 7G). Of interest, goblet cell hyperplasia was prevented when GMP was administered to animals suffering of FA (177.00 ± 19.58 cells/mm^2^; *p* < 0.001).

### 3.5. Oral Administration of GMP Down-Regulates the Type 2 Immune Response and Skews towards a Type 1 and Regulatory Profile

**Figure 8:**
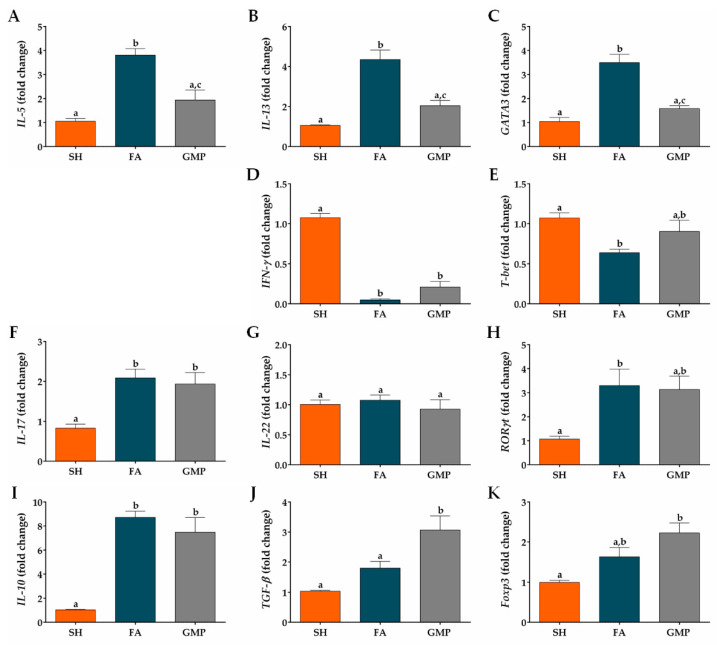
Effect of treatment with glycomacropeptide (GMP) on intestinal expression level of cytokines and transcription factors. Intestinal expression level of: cytokines (**A**) *IL-5* and (**B**) *IL-13* and transcription factor (**C**) *GATA3*, characteristics of type 2 immune response; cytokine (**D**) *IFN-γ* and transcription factor (**E**) *T-bet*, distinctives of type 1 immune response; cytokines (**F**) *IL-17* and (**G**) *IL-22* and transcription factor (**H**) *RORγt*, involved in type 17 immune response; cytokines (**I**) *IL-10* and (**J**) *TGF-β* and transcription factor master (**K**) *Foxp3*, inductors of regulatory immune response. Expression level of all cytokines and transcription factors relative to *β-actin* was analyzed by quantitative PCR and expressed as fold change (*n* = 4). SH, sham control; FA, FA-induced; GMP, FA-induced and GMP-treated animals. Different letters (a,b,c) indicate statistical difference.

Type 2 cytokines are involved in the pathogenesis of FA, being implicated in GI manifestations and alterations of intestinal mucosa [12,48]. Therefore, we investigated whether GMP suppresses the mRNA expression of *IL-5* and *IL-13* cytokines, as well as *GATA3*, a master transcription factor involved in type 2 immune response [49]. Expression level of *IL-5*, *IL-13* and *GATA3* were 3.6-fold, 4.1-fold and 3.4-fold augmented in animals of the FA group compared to SH animals (Figure 8A,B,C; *p* < 0.001). Prophylaxis with GMP in allergic animals remarkably reduced the gene expression of *IL-5* by 49% (*p* < 0.01), *IL-13* by 53% (*p* < 0.01) and *GATA3* by 55% (*p* < 0.001) at intestinal level. To further explore the effect of GMP on others cellular differentiation profiles at the small intestine microenvironment, the mRNA expression of different transcription factors and the main cytokines of each profile were evaluated. The expression of *IFN-γ* (Figure 8D) and transcription factor *T-bet* (Figure 8E) were significantly diminished in FA animals as compared to control rats (*p* < 0.0001 and *p* < 0.05, respectively). GMP treatment induces a slight increase in their expression, although it was not significant. Regarding the type 17 immune response, *IL-17* and *RORγt* mRNA expression was significantly augmented in intestinal tissue of FA animals (*p* < 0.01 and *p* < 0.05, respectively), and GMP treatment did not modulate this increase (Figure 8F,H). *IL-22* was not involved in intestinal inflammation of allergic animals (Figure 8G). In relation to the regulatory immune response, in our experimental model of FA the mRNA expression of *IL-10* was up-regulated (*p* < 0.001; Figure 8I) in comparison to sham animals, without a significant change in the level of *TGF-β* and *Foxp3* (Figure 8J,K). Strikingly, GMP treatment significantly increased the expression level of *TGF-β* in allergic animals (*p* < 0.05), and induced a slight augmentation in *Foxp3*, although it was not significant.

**Figure 9:**
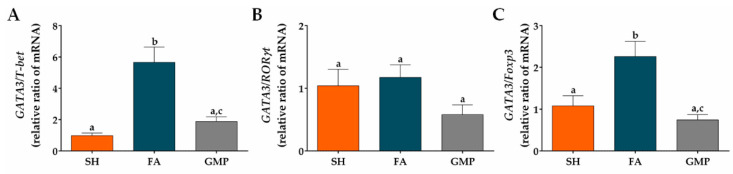
Effect of treatment with glycomacropeptide (GMP) on the skew of the immune response in the intestinal environment. Ratios of mRNA expression of (**A**) *GATA3*/*T-bet*, (**B**) *GATA3*/*RORγt* and (**C**) *GATA3*/*Foxp3* in intestinal tissue of experimental groups. Intestinal expression level of transcription factors relative to *β-actin* was analyzed by quantitative PCR and expressed as relative ratio of mRNA (*n* = 4). SH, sham control; FA, FA-induced; GMP, FA-induced and GMP-treated animals. Different letters (a,b,c) indicate statistical difference.

Finally, we calculated the *GATA3*/*T-bet* (type 2/type 1), *GATA3*/*RORγt* (type 2/type 17) and *GATA3*/*Foxp3* (type 2/regulatory) ratios to determine the degree of skew of the immune response in the intestinal microenvironment of allergic animals with or without GMP treatment. The rats with FA showed a significant bias to the type 2 response in the *GATA3*/*T-bet* and *GATA3*/*Foxp3* ratios, with respect to the response presented by sham animals (*p* < 0.01 and *p* < 0.05, respectively). GMP treatment reduced both *GATA3*/*T-bet* and *GATA3*/*Foxp3* ratios by approximately 67% in allergic animals (*p* < 0.01; Figure 9A,C), indicating a skew away from type 2 response and towards type 1 and regulatory responses. These data are in agreement with decreased *IL-5* and *IL-13* mRNA production, and increased *TGF-β* mRNA production in GMP-treated animals, as shown above (Figure 8A,B,J). The *GATA3*/*RORγt* ratio in intestinal tissue of animals with FA was not significantly modified by GMP administration (Figure 9B).

## 4. Discussion

Currently, there is no definitive treatment for FA. The avoidance of the food allergen, and the treatment with adrenaline or H2 receptor blockers are used to prevent or reduce symptoms [4,50]. Moreover, immunotherapy targets the underlying immune disorder, but it requires extended clinical visits, it is expensive and results are often temporary [51]. Thus, new strategies for preventing the FA development are needed.

In this study we investigate whether GMP attenuates GI manifestations, intestinal histopathological alterations and allergic immune response induced by oral challenges with the allergen in rats with FA. We chose Wistar rat as it has been reported as a strain that generates an adequate IgE response and the clinical manifestations of FA [16,17,18]. We first characterized an experimental model of FA with GI manifestations to OVA, based on reported protocols in which rats were systemically sensitized with the allergen combined with adjuvants or orally sensitized with the allergen and without adjuvants [16,38]. The rat model with systemic sensitization demonstrated allergen-specific IgE production, which is an immunological feature of FA [10]. Initial IgE production was later strengthened by oral challenges, showing that constant exposure to allergen does not induce development of oral tolerance in our experimental conditions. The production of anti-OVA antibodies of IgE isotype can be attributed to both aluminum adjuvant and *B. pertussis* which favor IgE production [52,53]. Animals also had increased histamine level in serum at day 27, with no changes in histamine or rMCP-2 values at day 40. In relation to anaphylactic response, after OVA intake animals presented cutaneous and GI manifestations, mainly diarrhea. These signs were accompanied with edema in the small intestine. Animals did not develop shock, as no change in rectal temperature was recorded. It has been reported that allergic diarrhea is mediated by mechanisms that are IgE and mast cell-dependent, and it is unaccompanied by signs of anaphylactic shock [12,54]. In our systemic sensitization model, the histamine and rMCP-2 amount released in serum by allergen-activated mast cells was not enough to develop anaphylactic shock, but locally induced GI manifestations. On the other hand, none of the aforementioned FA markers were present in animals from the oral sensitization model. The murine models of FA without adjuvants have been difficult to reproduce due to the induction of GI tolerance to food allergens, in addition to possible modifications of the allergen when passing through the digestive tract that can modify its allergenic potential [55,56,57]. Therefore, systemic sensitization with adjuvants and subsequent oral challenges are needed to develop a FA model with GI signs in Wistar rats.

GMP is a peptide derived from milk that exhibits biological activities with positive impact on human health [26]. In the present study, we demonstrated that prophylactic GMP administration induces a significant reduction in the development of FA by diminishing the IgE and IgG1 antibody response, the allergic clinical manifestations, and the intestinal inflammation. Prophylaxis with GMP decreases mast cell and eosinophil number in the intestine and causes a significant recovery on histopathological alterations of the intestinal mucosa, which are generated by the underlying chronic inflammation. Besides, oral GMP targets the intestinal type 2 inflammatory milieu by reducing *IL-5*, *IL-13* and *GATA-3* expression and skews the profile toward type 1 and regulatory responses, as it decreases *GATA3*/*T-bet* and *GATA3*/*Foxp3* ratios.

IgE is systemically produced during the induction or sensitization phase of FA and later is persistently generated and secreted in the tissue through local allergen challenge [58]. The induction of specific IgE antibodies is invariably accompanied by production of allergen-specific IgGs, such as IgG1, whose synthesis is also Th2 mediated [59,60]. Both IgE and IgG1 OVA-specific antibodies were produced in rats with FA, as previously reported in murine FA models [16,61,62]. While IgE levels progressively increased during systemic sensitization and oral boosters, the rapid increase in IgG1 to the maximum level was associated with oral allergen boosters. GMP treatment reduced the levels of OVA-specific IgE in serum over time and delayed the peak in OVA-specific IgG1. IL-13 is involved in IgE synthesis and TGF-β inhibits Th2 differentiation [63,64]. Previous reports show that oral GMP administration inhibits IL-13 and increases TGF-β production by splenocytes of allergen sensitized rats [28,65]. Thus, these systemic immune-modulatory effects of GMP might be mediating the IgE down-regulation during the development of FA. It has been described that higher amounts of type 2 cytokines are required to drive IgE synthesis than those that are sufficient for the synthesis of IgG1 [66]. The modulatory effects of GMP on the aforementioned cytokines may be enough to decrease IgE production, but only to alter IgG1 synthesis with a delay in reaching its maximum level. Besides, Monnai and coworkers demonstrated that in mice fed with a GMP supplemented diet, that ingested OVA as part of their diet and were systemically immunized with β-lactoglobulin in complete Freund´s adjuvant, the serum IgG level specific to both OVA and β-lactoglobulin was suppressed but IgE and IgA responses were unaffected [27]. Thus, the regulatory activity of GMP is falling on the predominant humoral immune response triggered systemically by the antigen.

IgE and IgG antibodies are linked to anaphylactic response in rodent models and in humans [67]. Animals treated with GMP decreased both IgE levels and signs of food anaphylaxis, showing that IgE is a key antibody involved in anaphylactic response in our experimental model. Besides, elevated histamine is associated with clinical signs during allergic reactions, mainly with GI manifestations [68,69]. Our results show that serum histamine was significantly lower in GMP-treated animals, which is in accordance with the lower severity of the anaphylactic response generated by oral allergen challenge. Apart from IgE, GI symptoms in food anaphylaxis also depend on mast cells [6]. The activation of mast cells by the allergen induces degranulation, with the release of preformed mediators, enzymes and cytokines, such as histamine, proteases and TNF-α [70,71]. Histamine induces vasodilation, increases vascular permeability and triggers edema formation [72]. This vasoactive substance has been associated with diarrhea in FA mice models [73]. TNF-α is involved in the recruitment of inflammatory cells such as neutrophils and eosinophils and has been recently found elevated in patients with FA [44,74]. On the other hand, IL-1 has been linked to the intestinal anaphylactic process, as the administration of an antagonist of IL-1 receptor in guinea pigs prevents allergen-challenge-induced colonic hypersecretion [75,76]. Additionally, there is a positive feedback loop between mast cells and IL-1, as IL-1 is able to induce histamine release from mast cells, and mediators released from these cells increase IL-1 production [77,78]. Thus, the low number of mast cells present at the intestine of GMP treated animals and the decreased expression of *TNF-α* and *IL-1β* might be causing the abolishment of diarrhea and intestinal edema after allergen challenge. These results are in agreement with the intestinal anti-inflammatory effect of orally administered GMP in experimental models of ileitis and NSAID enteropathy that is accompanied by a down-regulation on *TNF-α* and *IL-1β* expression [35,37]. As we have previously demonstrated that GMP inhibits mast cell activation and histamine release in response to allergen [65], a regulatory direct effect of GMP on mast cells cannot be disregarded. In addition, it is known that intestinal anaphylaxis in mice is accompanied by augmented gut permeability [12], similarly to the increased intestinal permeability presented by patients with FA [79]. *IL-1β* and *TNF-α* are involved in the alteration of intestinal barrier function, while TGF- β has been reported to preserve it [80]. Although we did not perform permeability assays, we believe that the inhibitory effect of GMP on *IL-1β* and *TNF-α* expression and the up-regulation of *TGF-β* at the intestine of FA animals may prevent an impaired intestinal barrier function, as it has been demonstrated with in vitro studies using cell lines of enterocytes and goblet cells [81,82]. This suggestion is supported by studies showing that GMP is able to inhibit signaling pathways involved in *TNF-α* and *IL-1β* gene expression, such as NF-κB and MAPK pathways, in the intestinal mucosa of mice with ulcerative colitis [83], as well as in cell lines of goblet cells [81], mature enterocytes [84], and macrophages [85]. Future research in this field will be necessary to demonstrate it.

Continuous oral exposure to the allergen induces intestinal histological changes that are associated with the late phase reaction of FA and with the intestinal chronic inflammation state [86]. In our FA animals, intestinal alterations were manifested by the reduction of the villi length, the increase of the crypts´ depth and the thickening of the internal *muscularis* layer. These changes were accompanied by eosinophil hyperplasia in the lamina propria and of goblet cells in the intestinal epithelium. A higher number of mast cells was also evidenced by the increase in *Mcpt2* expression. All these intestinal alterations are similar to those reported in other experimental models of FA [87,88,89]. Treatment with GMP prevented histopathological alteration in the intestinal tissue of FA animals. This beneficial effect of GMP might be causing the decrease in diarrhea severity, as McCallan and co-workers demonstrated a close association between intestinal damage and GI manifestations in children with FA [46]. It has been reported that mice over-expressing *IL-5* increase GI eosinophil number, with accumulation occurring within the lamina propria in the submucosa and villus [90]. Besides, IL-13 produced in intestine by inflammatory cells causes an increase in the amount of eosinophils and mast cells, and also goblet cell hyperplasia [91,92]. Thus, the decreased number of eosinophil and mast cells, and the reduction in goblet cell hyperplasia induced by GMP administration in FA animals are in line with the lessened level of expression of *IL-5* and *IL-13* in intestinal tissue. Our results are in accordance with the protective effect of GMP on the chronic inflammatory process that involves tissue remodeling in lung and skin associated with asthma and atopic dermatitis [32,33,93].

In order to establish the mechanism of action of GMP in FA, cytokines of type 1, 2, 17 and regulatory profiles, as well as their master transcription factors, were evaluated in the intestinal tissue. In FA, type 2 cytokines synthesized by Th2 cells and other immune cells are responsible for the intestinal allergic inflammation [11]. Apart from the aforementioned properties of IL-5, it plays a critical role in eosinophil differentiation, which is the main cause of intestinal eosinophilia associated with FA pathogenesis. Besides, it is involved in the activation and survival of eosinophils into mucosal tissues [94,95]. On the other hand, IL-13 is also an important regulator of the Th2 differentiation and IgE production [91,95,96]. The synthesis of both cytokines and the Th2 differentiation requires the participation of the master transcription factor GATA3 [49]. The dominant type 2 immune response in our FA group was represented by high expression level of *IL-5*, *IL-13* and *GATA-3*, as well as high *GATA3*/*T-bet* and *GATA3*/*Foxp3* ratios, as it is well-documented in experimental models and in humans [12,97,98]. FA development also decreased *IFN-γ* and *T-bet* expression and increased that of *IL-10*, *IL-17* and *RORγt* in intestinal tissue, as previously shown by others [12,99]. GMP administration significantly decreased *IL-5*, *IL-13* and *GATA3* expression in the intestinal tissue of FA animals, which is in accordance with reduced eosinophilia, goblet cell hyperplasia and IgE gene expression in small intestine of GMP treated rats. Anti-inflammatory activity of GMP in experimental asthma and atopic dermatitis has also been associated with decreased levels of *IL-5* and *IL-13* in affected tissues [32,33]. Immune-modulatory activity of GMP in small intestine of FA animals was also accompanied by a significant increase in *TGF-β* gene expression and a slight raise of *IFN-γ*, *T-bet* and *Foxp3* levels, that were evidenced with a marked down-regulation on *GATA3*/*T-bet* and *GATA3*/*Foxp3* ratios. TGF-β suppresses Th2 lymphocyte subset differentiation by inhibiting the expression of transcription factor *GATA3* [100]. In this line, exogenous TGF-β is able to suppress *GATA3* and up-regulate *IFN-γ* and *T-bet* expression in a FA model in transgenic mice, which is accompanied by a diminution of OVA-specific IgE levels and in the anaphylactic reaction to the allergen [101]. At the same time, anti-allergic effects of some probiotic bacteria are mediated by the shift of Th2 response to Th1 and T regulatory ones [98,102]. We previously described that oral administration of GMP to sensitized rats increases the amount of intestinal *Lactobacillus*, *Bifidobacterium* and *Bacteroides* and the production of TGF-β by allergen-stimulated splenocytes [65]. Taken together, our findings suggest that GMP protects on GI manifestations and intestinal alterations associated with FA, by shifting the immune balance towards the type 1 and regulatory profiles, and away from the type 2 response; and this effect might be mediated, at least partially, by stimulating the production of TGF-β at the intestinal level. Our results are supported by findings showing that in CD4+ T cells from FA mice GATA3 forms a complex with Foxp30 that prevents Foxp3 movement to *TGF-β* promoter locus [103]. When *T-bet* expression is increased, it binds GATA3 and dissociates Foxp3, which has the opportunity to move to *TGF-β* promoter. Our results are also consistent with previous studies showing that GMP increases Foxp3 expression in rat splenocytes, which favors the differentiation of T regulatory cells [104]. The description of the phenotype of the regulatory cell subpopulation that might be mediating immune-modulatory effect of GMP is the aim of our current research.

Studies about the immune-regulatory activity of GMP on other allergic conditions show an increased expression of *IL-10* in affected tissue by allergic inflammation, such as lung in asthma and skin in atopic dermatitis [32,33]. In our results, *IL-10* expression in the intestine of FA animals was not modified by GMP administration. It has been reported that IL-10 inhibits both the proliferation and the cytokine synthesis of Th2 cells [105]. The dominance of different regulatory cytokines in the control of type 2 inflammation by GMP may be due to the intrinsic characteristic of each tissue. Particularly, at intestinal level different cells are a TGF-β source to maintain a tolerogenic microenvironment and to induce the IgA synthesis, an important mucosal antibody with regulatory and protective functions [106,107,108]. *IgA* expression in intestinal tissue of FA animals administered with GMP showed a strong tendency to increase. Accordingly, in an experimental model of ulcerative colitis, GMP intake recovers the levels of secretory IgA in intestinal mucosa of mice and potentiates the anti-inflammatory signal transduction mediated by TGF-β [83]. GMP administration also results in an increased number of IgA-positive plasma cells in the intestinal lamina propria of healthy animals [109]. The role of this important mucosal immunoglobulin in FA is controversial, nevertheless it is suggested that it can restrict the quantity of the antigens in the GI mucosa and thus decrease the sensitization phase [110,111]. The level expression of *IgA* in intestinal tissue of the GMP treated rats might be influenced by TGF-β and IL-5, since TGF-β is a class-switching factor in B cells to produce IgA, and IL-5 induces the terminal differentiation of B lymphocytes into IgA-producing cells [112,113].

## 5. Conclusions

This study provides strong evidence that oral GMP can significantly down-regulate the allergic type 2 response of FA animals, which positively impacts on the attenuation of the GI manifestations, as well as intestinal inflammation and histopathological alterations. GMP may represent an effective and safe promising strategy for patients with FA, as there is not an established and effective cure that modulates the involved pathogenic mechanism.

## Figures and Tables

**Table 1:** Primers used in this study.

Target	Sequence	Access Number
*GATA3*	Fw: AGAAGGCAGGGAGTGTGTGA	NM_133293.1
	Rv: TTAGCGTTCCTCCTCCAGAG	
*Foxp3*	Fw: CGGGAGAGTTTCTCAAGCAC	NM_001108250.1
	Rv: CACAGGTGGAGCTTTTGTCA	
*T-bet*	Fw: TCCAAGTTCAACCAGCACCA	NM_001107043.1
	Rv: ATAAGCGGTTCCCTGGCATA	
*RORγt*	Fw: GCAGCAACGGGAACAAGTAG	XM_006232926.3
	Rv: GGGCTATACTCAAGGTGGCA	
*IgA-Fc*	Fw: CGGAACTATGAATGTGACCT	AJ510151.1
	Rv: GACTAAGGAGGGTTTTGGAC	
*IgE-Fc*	Fw: CGTCTGTCGGTTCTGATCTT	X00923.1
	Rv: GTCGCAGGATGAATGGAGTA	
*Mcpt2*	Fw: ATTATCGGTGGTGTGGAGTC	NM_172044.1
	Rv: GTGTGGATTCTCGCTTTCTC	
*IFN-γ*	Fw: GCCTAGAAAGTCTGAAGAAC	NM_138880.2
	Rv: GAGATAATCTGGCTCTCAAG	
*TNF-α*	Fw: GCCTCAGCCTCTTCTCAT	NM_012675.3
	Rv: CGCTTGGTGGTTTGCTACGA	
*IL-1β*	Fw: AAATCTCACAGCAGCATCTC	NM_031512.2
	Rv: ACTAGCAGGTCGTCATCATC	
*IL-5*	Fw: CAGTGGTGAAAGAGACCTTG	NM_021834.1
	Rv: GTATGTCTAGCCCCTGAAAG	
*IL-13*	Fw: ATCGAGGAGCTGAGCAACAT	NM_053828.1
	Rv: ATCCGAGGCCTTTTGGTTAC	
*IL-17*	Fw: CGTGAAGGTCAACCTGAAAG	NM_001106897.1
	Rv: TCTATCAGGGTCCTCATTGC	
*IL-22*	Fw: CTCTGCCCATCAACTCCCAA	NM_001191988.1
	Rv: TTGGCTTTGACTCCTCGGAA	
*β-Actin*	Fw: GTCGTACCACTGGCATTGTG	NM_031144.3
	Rv: GCTGTGGTGGTGAAGCTGTA	

Fc, fragment crystallizable.
